# Nanomaterial-Based Drug Delivery Systems for Pain Treatment and Relief: From the Delivery of a Single Drug to Co-Delivery of Multiple Therapeutics

**DOI:** 10.3390/pharmaceutics15092309

**Published:** 2023-09-13

**Authors:** Yuhang Xu, Xingpeng Dong, Heming Xu, Peifu Jiao, Lin-Xia Zhao, Gaoxing Su

**Affiliations:** 1 School of Pharmacy, Institute of Pain Medicine and Special Environmental Medicine, Nantong University, Nantong 226019, China; 2 School of Chemistry and Chemical Engineering, Qilu Normal University, Jinan 250200, China

**Keywords:** nanomedicine, drug delivery, pain treatment, combination therapy, nanoformulation

## Abstract

The use of nanomaterials in drug delivery systems for pain treatment is becoming increasingly common. This review aims to summarize how nanomaterial-based drug delivery systems can be used to effectively treat and relieve pain, whether via the delivery of a single drug or a combination of multiple therapeutics. By utilizing nanoformulations, the solubility of analgesics can be increased. Meanwhile, controlled drug release and targeted delivery can be realized. These not only improve the pharmacokinetics and biodistribution of analgesics but also lead to improved pain relief effects with fewer side effects. Additionally, combination therapy is frequently applied to anesthesia and analgesia. The co-encapsulation of multiple therapeutics into a single nanoformulation for drug co-delivery has garnered significant interest. Numerous approaches using nanoformulation-based combination therapy have been developed and evaluated for pain management. These methods offer prolonged analgesic effects and reduced administration frequency by harnessing the synergy and co-action of multiple targets. However, it is important to note that these nanomaterial-based pain treatment methods are still in the exploratory stage and require further research to be effectively translated into clinical practice.

## 1. Introduction

Pain is one of the most common global diseases adversely affecting human lives and exerts a considerable socioeconomic impact on the health care system and society. It is estimated that 20% of adults suffer from pain globally [1]. Currently, opioids are primarily used to manage and relieve pain. Notably, this single-drug treatment method has many disadvantages, such as that of many painkillers with side effects, sedation, liver toxicity, depression, respiratory depression, and addiction, among others [2]. These drawbacks are difficult to eliminate, influence the therapeutic effect, and cause many social problems. Therefore, improved therapeutic methods need to be developed for pain treatment.

The rapid development of nanotechnology in medicine and other fields has become a research hotspot in recent years. Nanoparticles (NPs) usually refer to particles with a particle size between 1 and 100 nm. They have unique physical and chemical properties due to their high surface area-to-volume ratio [3]. They can be categorized into two groups based on their composition: inorganic nanocarriers, mainly carbon nanotubes, mesoporous silicon nanomaterials, copper and gold NPs, and organic nanocarriers, including liposomes, polymer micelles, dendrimers, polymer conjugates, and polymer NPs [4,5]. As drug carriers, NPs have good biocompatibility and can effectively deliver drugs accurately to diseased tissues. Nanocarriers can improve therapeutic effects and treat diseases with minimal side effects by improving drug stability, reducing adverse reactions, controlling release efficiency, and changing pharmacokinetic characteristics [6]. Additionally, nanotechnology-enabled tools can enhance medical imaging techniques by providing better contrast agents and improved resolution [7,8]. Nanosensors and nanodevices allow for the real-time monitoring of various parameters within the body, providing valuable data for diagnosing and managing diseases [9]. The field of nanomedicine continues to evolve, and with ongoing research and advancements, it holds immense potential to transform how we diagnose, treat, and manage diseases, including pain management.

**Figure 1:**
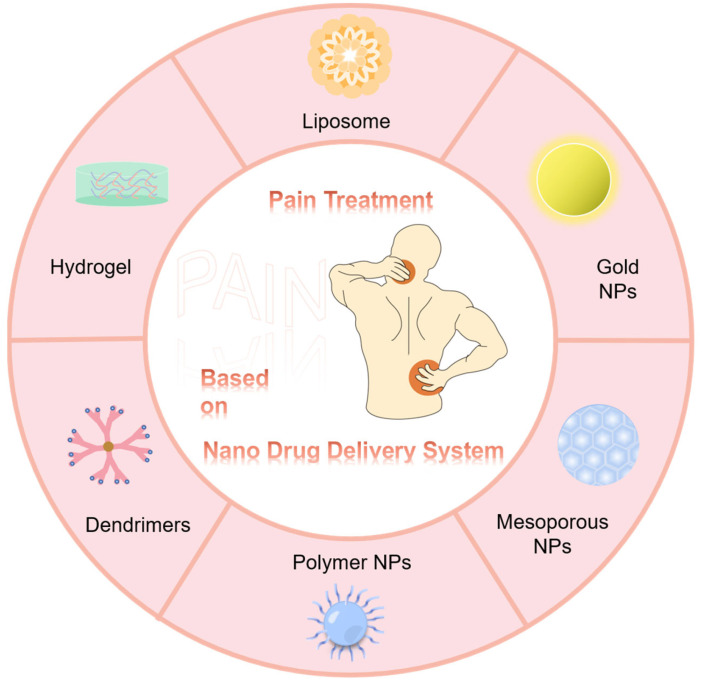
Popular nanomaterials are used as drug-delivery systems for pain treatment and relief.

NP-based drug delivery systems for pain treatment and relief have achieved numerous advances in preclinical research, providing a candidate strategy for accurate pain management (Figure 1). Additionally, they have a strong potential for the safer treatment of pain. This study reviewed pain treatment using single-drug or multi-drug combinations, focusing on the advances and challenges of nanoformulation-based pain treatment from single-drug delivery to multi-drug co-delivery.

## 2. Definition and Classification of Pain and the Common Drugs Used to Treat Pain

### 2.1. Definition and Classification of Pain

**Figure 2:**
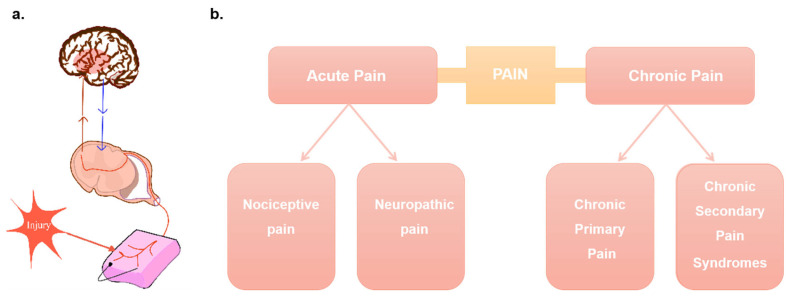
(**a**) Acute pain transmission pathway: Acute pain follows a three-neuron sensory pathway in which peripheral neurons travel from receptors through the dorsal root ganglion into the spinal cord. The spinal neurons then cross and rise through the spinothalamic tract to the thalamus, where they synapse with a third neuron and then reach the somatosensory cortex, generating a feeling of pain; (**b**) Pathophysiological classification of pain: Acute pain (no more than 3 months) and chronic pain (more than 3 months).

Pain is a complex physical and psychological activity defined by the International Association for the Study of Pain (IASP) (2023) as unpleasant sensory and emotional experiences associated with or similar to actual or potential tissue damage. It can be categorized into acute and chronic pain according to its course. Acute pain is often caused by an injury or a disease and follows a three-neuron sensory pathway in which peripheral neurons travel from receptors via the dorsal root ganglion into the spinal cord and synapse with gelatinous spinal neurons in the dorsal horn of the spinal cord. The spinal neurons then cross and rise through the spinothalamic tract to the thalamus, where they synapse with a third neuron and then reach the somatosensory cortex, generating a feeling of pain. Its duration is short, usually no more than 3 months [10,11]. Acute pain can be grouped into nociceptive pain and neuropathic pain. Nociceptive pain can be somatic pain or visceral pain, according to the location. Body pain is mainly related to the skin, muscles, and bones, and visceral pain is related to the chest, abdomen, and pelvis. Nociceptive pain is caused by actual or threatened damage to non-nervous tissue, and its transmission follows a three-neuron sensory pathway that recognizes pain once it reaches the somatosensory cortex. Neuropathic pain is caused by damage to nervous tissue in the central or peripheral nervous system. For example, a stroke can cause damage to central neurons, leading to neuropathic pain [12]. Chronic pain, which occurs in about 8% of the population, is long-lasting and occurs in the absence of tissue damage. Chronic primary pain, including chronic migraine, can often be considered a healthy condition. Chronic secondary pain syndrome is caused by other diseases, such as rheumatoid arthritis (RA) [13]. Chronic pain is a complex and multifaceted problem, and it is generally believed that inflammation of the peripheral and central nervous systems is the main factor in the pathogenesis of chronic pain. On the one hand, numerous non-neuronal cells exist in the peripheral nervous system, such as immune and glial cells, which are activated in the pain circuit of pain injury, causing local inflammation of the peripheral nervous system. On the other hand, central sensitization due to synaptic plasticity in the central pain pathway and increased neuronal reactivity due to pain injury lead to central nervous system inflammation. Usually, chronic pain does not disappear completely after treatment of the main cause (Figure 2). Additionally, acute pain extending beyond 12 weeks is considered as chronic pain [12,14].

### 2.2. Common Drugs Used to Treat Pain

In recent centuries, opioid analgesics have been employed to treat moderate-to-severe acute and chronic pain, and they are known for their potent analgesic effects. Opioid receptors are found in the central nervous system and peripheral tissues throughout the body, and their analgesic properties are mainly derived from the gene mucoid opioid receptor that encodes the μ receptor [15]. Commonly used opioid analgesics include morphine, tramadol, fentanyl, sufentanil, oxycodone, cannabidiol (CBD), β-caryoneen, enkephalin, demerol, methadone, codeine, pentazocine, and so forth. Local anesthetics, which mainly target sodium ion channels for the nerve block, are also used clinically to relieve pain. Anesthetics are classified into amide local anesthetics, such as propylcaine, lidocaine, mepivacaine, bupivacaine hydrochloride, and prorocaine. Ester local anesthetics include brucaine and benzocaine [16].

Nonsteroidal anti-inflammatory drugs (NSAIDs) produce analgesic effects mainly by inhibiting the function of cyclooxygenase (COX) isoenzymes and inhibiting the synthesis of prostaglandins. Common NSAIDs include aspirin, acetaminophen, ketoprofen, ibuprofen, naproxen, diflunira, nimesuride, indomethacin, diclofenac, leucophenol, meloxicam, celecoxib, and so forth. Compared with opioid analgesics, NSAIDs have a relatively weak analgesic effect. However, they have a lower potential for addiction, making their clinical use widespread [17].

In managing complex pain clinically, additional medications are often employed to enhance the treatment regimen. Epalrestat, a noncompetitive inhibitor of aldose reductase, can be used to inhibit diabetic peripheral neuropathy. Dexamethasone, a glucocorticoid, can inhibit inflammatory cells and has anti-inflammatory effects. Other pain medications include the calcium channel blocker ziconotide, sodium channel blocker tetrodotoxin, potassium channel openers nicodil and flupirtine, competitive antagonists of capsaicin receptor subtype 1, skeletal muscle relaxants tizanidine and baclofenac, antiepileptic drugs lamotrigine and escarbazepine acetate, β-adrenoceptor antagonist carvedilol, antifolate tumor drug methotrexate (MTX), antitumor drug temozolomide, selective 5-hydroxy tryptamine serotonin and norepinephrine reuptake inhibitor duloxetine, antihistamines promethazine and fexofenadine, anti-osteoporosis drug zoledronic acid, and anticonvulsant carbamazepine, among others [18,19,20].

### 2.3. Drug Combinations for Pain Treatment

The single-use of analgesics has many drawbacks, including weak therapeutic effects, the side effects of drugs, and problems in biological compatibility. Therefore, combining two or more drugs can often exert a better therapeutic effect with strong development potential. In recent years, drug combination in pain treatment has gained increasing attention. As early as 2014, a study reported that the combination of morphine and clonidine plays a synergistic role in the antinociception of mice [21]. Despite no synergistic effect in sedation and cardiovascular aspects, the dual drug combination has progressed in medicine. A team of researchers evaluated the effects of opioids and synthetic cannabinoids on mechanical pain caused by complete Freund’s adjuvants and streptozotocin, but this drug combination had little effect on inflammation [22]. In the same year, another study used a fixed-dose combination of tramadol/diclofenac for acute pain. The results showed that this combination not only alleviated severe pain (e.g., musculoskeletal pain and postoperative pain) but also significantly affected inflammation such as osteoarthritis (OA) [23].

Pecikoza et al. examined the interaction of the hypoglycemic drug metformin and duloxetine/oxycodone/eslicarbazepine acetate/vitamin B12 in relieving pain hypersensitivity in diabetes. The combination of metformin and these analgesics or vitamin B12 can be more effective in relieving pain and reducing the vitamin B12 deficiency caused by metformin [24]. Bedair investigated the therapeutic effect of the analgesic morphine combined with the association of tennis professionals-sensitive potassium channel opener nicodil in the rat model of hepatic fibrosis pain, revealing that this treatment strategy could significantly enhance pain resistance and have a significant hepatoprotective effect. Nicodil also improved morphine dependence in patients [25]. Chen et al. studied the antialgic effects and adverse reactions of flupirtine, a Kv7 potassium channel opener, combined with antihistamines promethazine and fexofenadine on acute and chronic pain in mice. This study established a model of writhing body pain induced by acetic acid, inflammatory pain induced by carrageenan, and neuropathic pain induced using paclitaxel. Antihistamines play a synergistic analgesic role by activating Kv7/M channels [26]. CBD and beta-caryophyllene (BCP), the non-psychoactive components of cannabis, have more benign side effects than other cannabis components and can reduce neuropathic and inflammatory pain. The combined analgesic potential of CBD and BCP in a rat model of chronic pain caused by spinal cord injury was evaluated. When co-administered in a fixed ratio, CBD and BCP caused an enhanced dose-dependent reduction, with a synergistic effect observed in cold hypersensitivity in both sexes and an additive effect observed in tactile hypersensitivity in men. These findings suggested that the combined administration of CBD/BCP might provide a safe and effective treatment option for treating chronic spinal cord injury [27]. Li et al. investigated DAMGO-NH_2_, an amide analog of the opioid receptor agonist [D-Ala^2^, NMe-Phe^4^, Gly^5^-ol]-enkephalin (DAMGO) to address the issue of drug tolerance in combination with the peripheral limiting nociception/orphanin FQ (N/OFQ) receptor (NOP) peptide agonist NOP01, and investigated the antialgic effects of this combination, which showed lower tolerance and side effects compared with a single dose [28]. The aforementioned research results showed that the treatment model of drug combination profoundly changed the pain treatment landscape.

## 3. Single Drug-Based Nanoformulations for Pain Treatment

Over the years, various NPs have been developed and skillfully used to deliver local anesthetics, NSAIDs, and opioids (Table 1). Considering the precise surface modification and encapsulation properties of NPs, analgesics can achieve higher solubility and half-life in nanocarriers and allow controlled release, thus improving pharmacokinetics and biodistribution. Additionally, nanodrug delivery systems can also reduce the frequency of administration and the body’s barrier to the drug, thereby reducing its side effects and improving efficacy. In conclusion, applying nano-borne drugs provides substantial efficiency and safety for analgesia [29,30].

### 3.1. Liposomes

Liposomes are closed vesicles composed of nontoxic cholesterol and phospholipids, which can encapsulate hydrophilic or hydrophobic drugs and can be integrated with cell membranes, good biocompatibility and biodegradability, low toxicity, high loading capacity, and large retention and permeability [31]. In the last 30 years, different optimized liposome particles, such as cationic liposomes, heat-sensitive liposomes, and pH-sensitive liposomes, have been fabricated by modifying the liposome surface through various engineering strategies. Liposomes as drug carriers exhibit significant potential [32].

As early as 2006, a study showed that the use of liposomes containing three local anesthetics, prilocaine, lidocaine, and mepivacaine, for the suborbital nerve block test not only improved the half-life and bioavailability of the drugs in rats but also improved the bioavailability of the drugs after the delivery of liposomes, compared with the direct injection of these three local anesthetic liquids. Among these, mepivacaine had the remarkable sealing effect and the best efficacy among the three liposome nanoformulations because of its high allocation coefficient [33]. Bupivacaine-loaded liposomes were also prepared to treat pain in recent years. Additionally, the adjuvant use of bupivacaine-loaded liposomes for lumbar fusion surgery might promote early activity, reduce opioid consumption, and shorten hospital stays, thereby reducing overall healthcare costs [34].

### 3.2. Solid Lipid NPs

Unlike traditional liposomes with poor physical stability, the first-generation lipid NPs are composed of solid lipids called solid lipid NPs (SLNs). SLNs were used as a topical drug delivery carrier to carry anti-inflammatory drugs ketoprofen and naproxen. The release time of SLNs in the epidermis was extended due to their potential targeting effect and the properties of controlled release in different skin layers; the drug can accumulate in the stratum corneum, thus achieving anti-inflammatory effects [35]. Recently, Vishwakarma et al. delivered epalrestat with SLNs and evaluated their effects on diabetic neuropathic pain in a rat model. The results showed that the SLNs synthesized using the micro emulsification method had good encapsulation efficiency and were stable at acidic stomach pH, which was suitable for oral administration. Compared with epalrestat standard treatment, epalrestat with SLNs had the same efficacy, was less hepatotoxic, and could effectively improve streptozotocin-induced diabetic neuropathic pain [36].

The second-generation lipid NPs, also known as nanostructured lipid carriers (NLCs), are composed of solid lipids and a certain amount of liquid lipids, with improved encapsulation rate (99.5%), increased storage stability, and better tolerance in vivo. NLCs were designed and developed to optimize the formulation of butyl-substituted benzocaine analog butamben to solve the local intoxication caused by local anesthetics. This system not only significantly increased the solubility of butamben but also reduced its in vitro cytotoxicity and in vivo toxicity and significantly improved the analgesic effect [37].

### 3.3. Dendrimers

Dendritic macromolecules comprise an inner core of multiple branching functional groups. There are various ways to generate dendritic macromolecules. The most common method is divergent synthesis, where one or more reaction points in the central nucleus react units with branching structures to obtain the first generation of molecules. Transforming the functional groups at the end of the first-generation molecular branch into functional groups can continue the reaction and then repeat the reaction with the branch unit reactant to obtain the second-generation molecule. Continuously repeating the above two steps can result in the desired dendritic macromolecule. They have a highly branched macromolecular structure, and the exact number, size, and structure of functional groups are highly controllable. The dendrimer macromolecules can self-assemble to form stable single-molecule micellar nanocarriers in solution, which avoids the decomposition of amphiphilic polymers under physiological conditions and has a high drug-loading capacity [38]. The most common dendrimers include poly(amidoamine) (PAMAM) dendrimers, polypropylene dendrimers, polyesters, and triazines. Besides their external surface functional groups, these dendrimers can be coupled to different molecules. The conjugation, whether covalent or non-covalent, with drugs determines their use [39].

Compared with a traditional preparation, dendrimer is a highly efficient solubility enhancer of NSAIDs. The mechanism of solubility enhancement is closely related to its structure, with its larger central core, branch units providing weak hydrogen bonds, and surface groups providing electrostatic action to increase solubility. Koc et al. investigated the solubility of PAMAM dendritic macromolecules loaded with NSAIDs (ketoprofen, ibuprofen, and diflunira) in a buffer solution to improve the low solubility and low bioavailability of NSAIDs. The results showed that the solubility enhancement performance of hydrophobic drugs in PAMAM dendritic molecules was four times that of ethylenediamine-cored PAMAM dendritic macromolecules due to their novel polypropylene oxide nuclei. Therefore, the application and optimization of dendrimer carriers have significant potential and application prospects in treating pain-induced inflammation [40].

### 3.4. Poly(Lactic-co-Glycolic Acid) NPs

Poly(lactic-*co*-glycolic acid) (PLGA) is a polymer randomly polymerized from lactic and glycolic acids. Different proportions of monomers can produce different PLGAs, and their degradation degrees are different. PLGA has attracted attention due to its unique characteristics of simple preparation, biodegradability, and high drug-loading capacity. The slow release of drugs encapsulated in PLGA can reduce routine dosing, enhance patient compliance, reduce discomfort, and avoid side effects [41]. Han et al. developed biocompatible and biodegradable ketamine-loaded polyethylene glycol (PEG)–PLGA for sustained release to overcome the short half-life of the analgesic adjunct ketamine in treating pain. The drug-carrying nanoparticles exhibited sustained release properties for up to 21 days in vitro and for more than 5 days after intravenous injection in mice [42]. Another study loaded the anti-aging drug ABT263 into PLGA NPs and obtained PLG(PEG)A-ABT, which was administered intraperitoneally to a rat model of injury-induced disk degeneration. The drug delivery system effectively removed senescent cells in the intervertebral disk, reduced the expression of pro-inflammatory cytokines and matrix proteases, inhibited the progression of intervertebral disk degeneration, and even restored the structure of the intervertebral disk structure [43]. Given the advances in nanodrug delivery technology in clinical applications, PLGA-based NPs have the potential to become unique and beneficial controlled release agents in the future.

### 3.5. Hydrogels

Hydrogels are a class of extremely hydrophilic three-dimensional network structure gels, which swell rapidly in water and can hold a large volume of water without dissolving in the swelling state. Hydrogels can be formed from either water-soluble or hydrophilic polymers through certain chemical or physical crosslinking. They have been extensively studied over the past 60 years and have shown great promise as a biocompatible material in many therapeutic applications [44].

**Figure 3:**
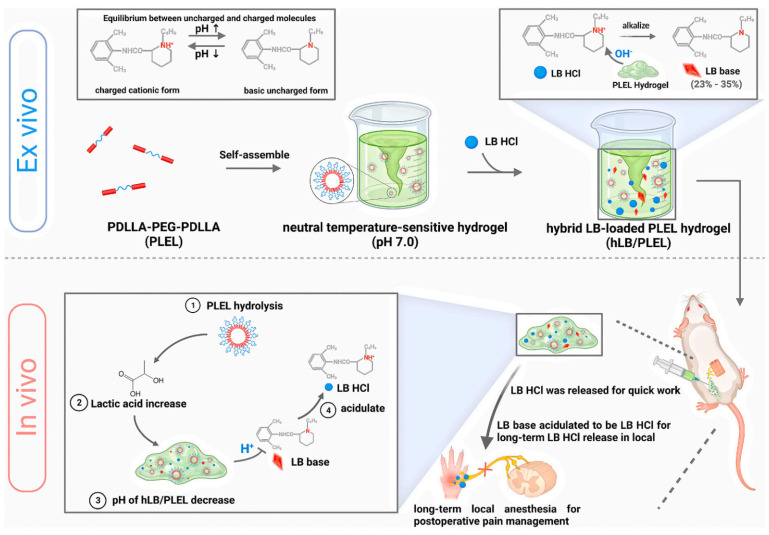
Schematic illustration of hybrid levobupivacaine-loaded temperature-sensitive injectable hydrogel system used for the sustained release of anesthetics to achieve long-term local anesthesia in postoperative pain treatment [45]. Copyright 2023 Elsevier.

Local anesthetics are clinically preferred for postoperative pain treatment to reduce the occurrence of a series of postoperative problems. However, the action time of local anesthetics is extremely short. Therefore, a temperature-sensitive hydrogel system was studied, a liquid that could flow at room temperature and was transformed into semi-solid hydrogels once it entered the body. The hydrogel system not only lasted six times longer than ordinary levobupivacaine acid but also effectively relieved spontaneous pain in a rat model of sciatic nerve block, subcutaneous infiltration anesthesia, and postoperative pain (Figure 3) [45]. Oh et al. found that the hydrogel device not only significantly enhanced the release rate of local anesthetics such as ropivacaine but also extended the pain relief time of the drug to 24 h or more. Therefore, the temperature-controlled hydrogel significantly reduced the toxicity of local anesthetics and had a lasting impact in the field of local anesthesia [46,47].

### 3.6. Mesoporous Silica NPs

Mesoporous silica NPs (MSNs) comprise a honeycomb porous structure with uniform, adjustable, and orderly pore sizes. Mesoporous silicon material has a large surface area and abundant silanol groups on its surface. Therefore, both the inner and outer surfaces of the carrier material, including an inner cylindrical pore and an outer granular surface, can be attached or modified by functional groups. This not only improves the stability and redispersability of the material but also enables targeted and controlled delivery of the therapeutic agent to a specific site in the body. Compared with traditional nanomaterials, mesoporous silicon material has higher physicochemical stability and biosafety and hence is a promising drug and gene carrier [48,49].

Starting from the mechanism of pain, to inhibit δ-opioid receptor (DOPr), a research team selected DOPr agonist encapsulated in MSNs, which was released in an acidic environment and reduced in vivo environment to inhibit the excitation of primary sensory neurons, spinal cord, and supraspinal neurons, thereby inducing analgesia. The results of this study supported the hypothesis that DOPr endosomal signaling was an endogenous mechanism and therapeutic target for relieving inflammatory pain and provided a new idea and channel for pain treatment [50].

MSNs have certain advantages in delivering insoluble drugs because of their special mesoporous properties. When insoluble drug molecules are encapsulated in mesoporous silica, their crystallization is minimized due to spatial confinement within the mesopores. The amorphous form of the drug, in contrast to its crystalline counterpart, exhibits reduced lattice energy, leading to an enhanced dissolution rate. Additionally, the exceptionally high hydrophilic surface area of mesoporous silica assists in the wetting and dispersion of the drug, promoting rapid dissolution [51]. Oral NSAIDs nimesulide and indomethacin are less water soluble and have poor bioavailability. Gou et al. packaged these drugs in carboxylated mesoporous silicon materials with high specific surface area. The carboxylated drug carrier had good in vitro release performance, besides the potential pH response release effect. The bioavailability of this system significantly improved, having excellent application prospects in anti-inflammatory and analgesic research [52].

### 3.7. Other Nanoformulations

Kopach et al. tested the therapeutic potential of nano-engineered microcapsules for pain treatment due to the poor pharmacokinetics of a single injection. The nano-engineered microcapsules are prepared using layer self-assembly technology. The polyelectrolytes with opposite charges are alternately adsorbed on the template to prepare the multilayer membrane structure. The sodium channel blocker QX-314 was encapsulated in biodegradable microcapsules made of poly-L-arginine and dextran sulfate to establish a rodent model of peripheral inflammation. The results demonstrated that the microcapsules biodegraded within a few weeks. The overall therapeutic effect lasted 10–20 times longer than a single local injection of the drug [53]. He et al. found that when drugs were administered directly through intra-articular injection, they were cleared from the joint space. Hence, subsequent drugs must be injected multiple times, thus causing systemic poisoning. To address this issue, He et al. designed a cationic polybrachyavidin nanostructure, which contained 28 covalent drug coupling sites and carried dexamethasone to enable continuous controlled release at therapeutic doses over several days. The results showed that a single low dose of nanodrug could effectively inhibit the inflammatory response and was significantly better than an unmodified dexamethasone injection [54]. Recently, a study established an animal model of neurogenic inflammatory pain. Borneol-modified liposomes were fused with mesenchymal stem cell exosomes, and ziconotide was loaded to prepare microneedles, which could significantly improve the efficiency of ziconotide crossing the blood–brain barrier. This system can be used safely and effectively to treat chronic pain and has significant potential for clinical application [55].

**Table 1:** Single-drug delivery systems to treat pain.

Drugs	NPs	Route of Administration	Pain Type	Therapeutic Effects	Reference
Prilocaine Lidocaine Mepivacaine	Liposomes	Rat (suborbital injection)	Postoperative pain	Liposomal encapsulation had a significant effect on mepivacaine and the least effect on lidocaine, possibly due to its remarkable vasodilatory properties	[33]
Bupivacaine	Liposomes	Adult patients with lumbar spinal fusion (intramuscular injection)	Pain during lumbar fusion surgery	Adjuvant use of the combination during lumbar fusion surgery may promote early activity, reduce opioid consumption, and shorten hospital stays, thereby reducing overall healthcare costs	[34]
Epalrestat	SLNs	Rat (oral)	Diabetic neuropathic pain	Epalrestat-SLN is equally effective and has less liver toxicity Compared with traditional epalrestat, it can effectively improve diabetic neuropathic pain induced by streptozotocin	[36]
Butamben	NLCs	Franz diffusion cells/adult male Wistar rats (injection into the posterior sciatic nerve area of the knee)	Inflammatory pain	The preparation showed potential for treating inflammatory pain, showing significant analgesic (40%) and long-lasting effects	[37]
Ketoprofen Ibuprofen Diflunisal	PAMAM		Inflammatory pain	The solubility of NSAIDs is directly proportional to the concentration and production of dendrimers The dendrimer is a highly effective solubility enhancer for NSAIDs due to its novel polypropylene oxide nuclei	[40]
Ketamine	PLGA	Mice (intravenous injection)	Neuropathic pain	Ketamine-loaded PEG-PLGA NPs prepared using this new nanoprecipitation method could achieve high drug load and slow-release properties	[42]
ABT263	PLGA	Rat (intervertebral injection)	Chronic low-back pain	This strategy could selectively eliminate senescent cells in degenerative intervertebral disks, reduce the expression of pro-inflammatory cytokines and matrix proteases in intervertebral disks, and even restore the structure of intervertebral disks	[43]
Levobupivacaine	Hydrogels	HT22 and C2C12 cells/rat (injected around the sciatic nerve)	Postoperative pain	The system could not only achieve rapid effect but also continuously release levobupivacaine for blocking nerves, significantly prolonging the local analgesic effect; it is an ideal choice for long-term postoperative pain treatment	[45]
Ropivacaine	Hydrogels	Sprague Dawley (SD) rat (subcutaneous injection)	Surgical wound pain	Local administration of hydrogels might be an effective way to prolong pain relief, with advantages including reduced systemic side effects and high localization of the drug in the target tissue	[46]
Ropivacaine	Thermosensitive hydrogel	Mice (subcutaneous injection)	Postoperative pain	The system had a small initial burst release, long-term nerve block, and good biocompatibility in vitro and in vivo and could be used for continuous local anesthesia without systemic toxicity	[47]
DADLE	MSNs	Human embryonic kidney 293 cell line /mice/patients	Inflammatory pain	The NPs caused persistent activation of DOPr in the endosome, which continuously inhibited the excitability of nociceptors and relieved inflammatory pain	[50]
Nimesulide /indomethacin	MSNs	SD rat (oral)	Inflammatory pain	MSN-COOH had good in vivo and in vitro administration performance for NSAIDs, including nimesulide and indomethacin, and the relative bioavailability is 3.6 times and 2.0 times higher than that of single drug, respectively. It has potential research value for the anti-inflammatory and analgesic effects of NSAIDS.	[52]
QX-314	Nano-engineered microcapsules	Wistar rats (subcutaneous injection)	Peripheral inflammation	Nano-engineered envelopes provide localized drug delivery suitable for long-term pain relief	[53]
Dexamethasone	Multi-arm Avidin nano-construct	Cartilage explants (intra-articular injection)	OA	The nanostructures had high conversion potential, enabling a single low-dose drug injection to treat OA and eliminating the toxicity issues associated with multiple high-dose injections	[54]
Ziconotide	Microneedle	Rat (tail vein injection)	Peripheral nerve injury, diabetes-induced neuropathy pain, chemotherapy-induced pain, and ultraviolet-B radiation-induced neurogenic inflammatory pain	This nano-loaded drug combination could effectively penetrate the skin to release drugs and had obvious analgesic effects on different pain models	[55]

## 4. Drug Combination-Based Drug Delivery Systems for Pain Treatment

Nanocarrier-based drug co-administration technology combines two drugs in an improved drug delivery system to achieve synergistic analgesia. Compared with the direct co-administration of free drugs, the co-loading of nanocarriers has several advantages. In these systems, the co-encapsulated drugs do not interfere with each other by loading at different regions of nanostructures (i.e., different layers, core or shell, and inner or outer), and the dosages of different drug components can also be flexibly regulated, significantly reducing side effects and having a synergistic effect. Further, the multi-drug delivery system can control the time of multi-drug action so as to achieve the continuous action of drugs. Briefly, the nanomaterial-based multi-drug delivery systems can realize synergistic multi-pathway and multi-target effects. The development of drug combinations based on nanodrug delivery technology in pain treatment is summarized in Table 2.

### 4.1. Liposomes

Liposomes, as the first batch of particles to be applied in nanomedicine, were applied to treat pain with dual drugs in 2009. Elron-Gross et al. used a liposome-carrying hyaluronic acid as a surface-anchored ligand and simultaneously containing dexamethasone and diclofenac and delivered them to the diseased site in rats with OA. This study found that two different drug packs were contained in the same liposome without interfering with each other and had fully retained biological activity. It overcame the serious adverse reactions associated with oral NSAIDs in the traditional treatment of chronic OA, including gastrointestinal toxicity and allergic reactions. The combined drug delivery treatment was significantly more effective, and the inflammatory volume was reduced considerably compared with the single dose or untreated group [56]. Sodium or calcium channel blockers are often used to treat localized neuropathic pain. Tetrodotoxin, a sodium channel blocker, and capsaicin, the active ingredient in chili peppers, can produce sense-selective peripheral nerve blocks, and a combination of the two can have a synergistic effect on the duration of the nerve blocks, thereby reducing postoperative pain. The combination of two drugs encapsulated in liposomes and injected into the sciatic nerve of male rats showed minimal myotoxicity and muscle inflammation, with no systemic toxicity [57]. Franze et al. also designed a lidocaine/CBD mutable liposome-based delivery system, targeting pain in sodium and calcium channels, where the addition of nanocarriers increased skin permeability and enabled drug delivery to deeper layers of the skin. The preparation is structurally stable and can be stored at 25 °C for 14 months. [58]. These studies provided new insight for developing more effective local anesthetics for pain treatment and other applications.

### 4.2. NLCs

In dental practice, local anesthetics are used to reduce discomfort caused by needle insertion and injection and to reduce symptoms of superficial trauma to the oral mucosa; however, no proven commercial formulation is available. Some studies optimally prepared NLCs, and lidocaine (59%) and prilocaine (66%) were co-encapsulated in the carrier, confirming that the lipid structure did not change due to the local anesthetic encapsulation and the formulation had a long storage time and satisfactory slow-release characteristics [59]. In anti-inflammatory therapy, the researchers prepared NLCs loaded with celonol and indomethacin (CEL-INdo-NLCs), and the transdermal administration of CEL-INdo-NLCs was performed. No skin irritation and gastrointestinal dysfunction were observed in rats after CEL-INdo-NLCs administration by regulating the levels of interleukin-1ꞵ (IL-1β), tumor necrosis factor-α (TNF-α), and B-endorphin, and no renal and reproductive toxicity was noted. The results showed that the drug delivery system could reduce plantar edema and inhibit inflammation and pain [60]. Yuan et al. optimized the NLC-based drug delivery technology. They designed a transdermal nanocarrier modified with a transactivator of transcription (TAT) peptide to contain ropivacaine, a local anesthetic, and meloxicam, a long-acting NSAID. The anesthetic analgesic ability of the system significantly improved after TAT modification, the skin penetration efficiency increased, the inflammation in the injured area was reduced, and pain treatment also improved. It provided an efficient and simple method for preparing this nanosystem and was expected to be a synergistic analgesic system [61].

### 4.3. PLGA NPs

Lee et al. developed multilayer lidocaine and adrenergine-eluted biodegradable PLGA nanofibers using electrospinning technology, which could sustainably elute hemostatic and analgesic drugs at oral wound sites, indicating that they had a good hemostatic effect and sustained pain relief, and could be used for the initial healing of palatine oral wounds [62]. Kao et al. used electrospinning technology to construct a PLGA nanofiber membrane for surgical wounds loaded with sheath structure anesthetic lidocaine and human epidermal growth factor; also, they evaluated their effectiveness in relieving pain and promoting healing. The results showed that this system could overcome problems such as intestinal obstruction, infertility, chronic pelvic pain, and abdominal pain caused by postoperative adhesion [63]. Furthermore, PLGA-based nanoformulations were developed to overcome the brain-blood barrier for neuropathic pain treatment. Nigam et al. developed the nasal delivery of baclofenac and lamotrigine to the brain via PLGA NPs for treating neuropathic pain. Baclofenac could significantly reduce the action of the excitatory neurotransmitters aspartate and glutamate. Lamotrigine acts by blocking sodium ions to maintain the membrane potential between neurons, thereby inhibiting the release of glutamate. The combination of these two drugs can be used to relieve pain. When they were co-encapsulated in PLGA, repeated use of the drug was avoided, significantly reducing the peripheral organ toxicity that may occur with repeated doses of lamotrigine alone. In vivo studies showed that this delivery combination reached the brain and stayed there for a longer time, with potential effects on inflammatory pain [64].

**Figure 4:**
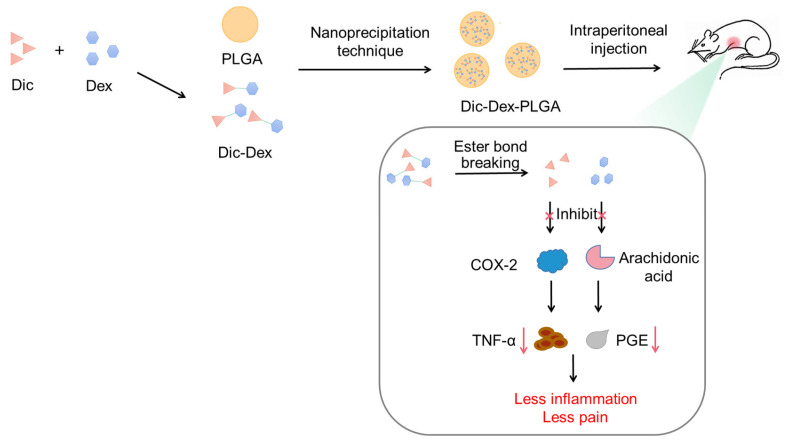
Dexamethasone–diclofenac-supported PLGA NPs inhibited the inflammatory process.

PLGA NPs are also widely used for treating inflammation. Dexamethasone has anti-inflammatory activity, but the long-term use of dexamethasone can cause bone damage and joint rupture. Additionally, the combination of diclofenac and dexamethasone reduces the dose of both drugs and alleviates the side effects that exist with a single drug. Both drugs are hydrophobic and have low water solubility. Assalia et al. prepared carvedilol-loaded polylactic acid for the first time, and the system showed higher water solubility and slow-release behavior, overcoming the limitations of the combination of two drugs (Figure 4) [65]. The combination of corticosteroids and NSAIDs is also commonly used clinically for inflammation and chronic joint pain. As mentioned earlier, the long-term use of these two types of drugs can lead to knee osteonecrosis. A PLGA microsphere microcrystalline gel delivery system loaded with dexamethasone and celecoxib was designed for intra-articular injection to overcome these unmet medical needs. Its biological activity was studied in a macrophage inflammatory model and a rat model of monomer sodium iodoacetate–induced OA. The results indicated that this treatment method protected the cartilage from erosion and reduced the release of pro-inflammatory cytokines. It not only reduced the potential stimulation of celeoxib but also maintained the release of dexamethasone, displaying remarkable effects on the treatment of OA through different functions and mechanisms [66].

### 4.4. Hydrogels

**Figure 5:**
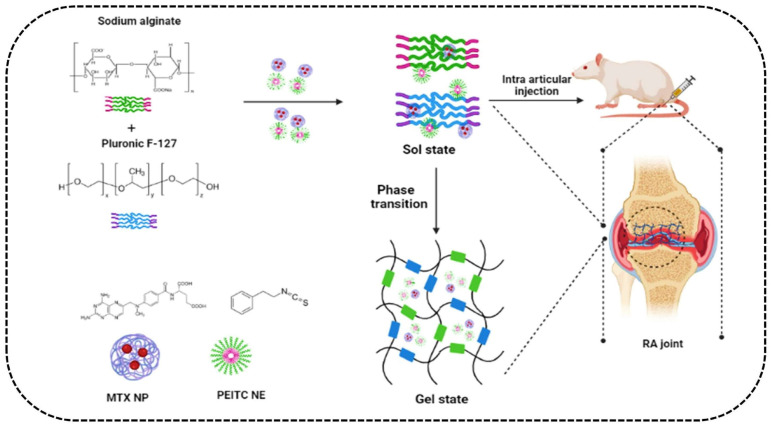
A schematic illustration of the preparation for treating RA using dual-drug NP-loaded hydrogel [70]. Copyright 2023 Taylor & Francis.

In recent years, analgesia through the nerve-block delivery of local anesthetics has been a popular method to reduce postoperative pain. However, local anesthetics always have the drawbacks of short half-life and strong systemic toxicity. In order to prolong the activity of local anesthetics, some studies have shown that local anesthetics need to be used in combination with nerve blockers such as dexamethasone. However, the local anesthetic ropivacaine without nanocarriers often causes inflammation in the body. Chitosan thermogel has been used as a drug carrier to embed ropivacaine and dexamethasone at the same time for clinical postoperative analgesia. It is effective in vivo for up to 48 h and can be degraded over time [67]. Zhang et al. performed similar research, confirming that the formulation could be used as a promising local anesthesia system. Gel delivery systems still have significant potential for tackling inflammation [68]. Studies were conducted to prepare and characterize a gel-based administration of ibuprofen and capsaicin, thereby inhibiting cyclooxygenase action and reducing prostaglandin synthesis, which could improve the anti-inflammatory and analgesic effects of these two drugs in vivo [69]. Similarly, Haloi et al. recently developed a hydrogel to co-deliver MTX and phenethyl isothiocyanate (PEITC) for treating RA. The solubility study showed that the nanocomposite hydrogel system increased the solubility of the two drug carriers to 73.13 ± 8.86% (29.25 ± 3.55 µg/mL). Additionally, the rat ankle inflammation model showed that this double-heavy drug system downregulated the expression of inflammatory factors and even almost completely restored the ankle morphological characteristics of rats. It achieved good anti-inflammatory activity and has high research value (Figure 5) [70].

### 4.5. MSNs

The dual surface of MSNs allows the delivery of different drugs in a multi-stage manner, which may be ideal for targeting chronic pain. Xie’s team designed a mesoporous silicon material that simultaneously contained the cannabinoid Δ9-tetrahydrocannabinol (Δ9-THC) and the erythropoietin-derived polypeptide cibinetide. THC dispersed spontaneously into the circulation, and the cibinetide peptide was released after glutathione-triggered disulfide bond breaks. Both had analgesic and anti-inflammatory effects. Preliminary studies showed that the drug delivery system could continuously release analgesic molecules and was highly safe, thus effectively inhibiting chronic neuropathic pain. This provided evidence for the feasibility of its application in treating neuropathic pain, which might help promote the development of effective treatments for chronic pain [71].

### 4.6. Other Nanoformulations

Meloxicam is a drug used for treating RA and postoperative pain. However, the oral effect is poor, the bioavailability is low, and the gastric damage is substantial. Tizanidine not only has obvious analgesic and anti-inflammatory effects but also enhances the analgesic and anti-inflammatory effects of meloxicam and can improve gastrointestinal tolerance. Therefore, some studies reported the preparation of meloxicam and tizanidine in a double-layer oral membrane. The double-layer oral membrane was divided into a slow-release layer and a quick-release layer, which released tizanidine not only quickly but also in a controlled and sustained manner. This combination of drugs increased plasma concentration and half-life and improved patient compliance. Studies showed that this drug delivery system could be used to treat various pain conditions, with significant potential [72]. Further, a polymer wafer containing ketorolac acid/lidocaine was developed to reduce pain and discomfort after gingival resection and to promote wound healing [73].

**Table 2:** Nanocarrier-based dual drug therapy for pain treatment.

Dual Drug Combination		NPs	Route of Administration	Pain Type	Therapeutic Effects	Reference
Drug A	Drug B					
Dexamethasone	Diclofenac	Liposomes	Rat (intra-articular injection)	OA	Both drugs retained their biological activity in the same liposome, and the combination reduced the inflammatory volume from the initial dose to 12.9%	[56]
Capsaicin	Tetrodotoxin	Liposomes	Rat (injected into the sciatic nerve)	Postoperative pain	Tetrodotoxin encapsulation combined with capsaicin significantly extended nerve block time to 18.2 h, much higher than capsaicin liposomes and tetrodotoxin liposomes. This combination caused minimal myotoxicity and muscle inflammation, with no systemic toxicity.	[57]
Lidocaine	Cannabidiol	Liposomes	Franz diffusion cells/porcine ear skin (injected into the skin)	Neuropathic pain	The drug delivery system could more effectively contain drugs in the dermis, and was more effective at improving drug skin penetration than control preparations carrying free drugs	[58]
Lidocaine	Prilocaine	NLCs	Franz diffusioncells	Postoperative pain	The lipid structure was formed, which remained unchanged after local anesthetic encapsulation and was amorphous within the lipid matrix The formula was stable at room temperature for 14 months and had satisfactory slow-release properties for lidocaine (59%) and prilocaine (66%) within 20 h	[59]
Celastrol	Indomethacin	NLCs	Franz diffusion cells/rat (injected into the skin)	RA	It showed a significant reduction in plantar edema, inhibition of inflammation and pain, and no skin irritation and renal toxicity	[60]
Ropivacaine	Meloxicam	NLCs	SD rat (applied on the skin surface)	Postoperative pain	After TAT modification, the analgesic ability of the system was improved by reducing inflammation in the injured area through improved skin penetration efficiency and co-delivery of the two drugs	[61]
Lidocaine	Epinephrine	PLGA/collagen nanofibers	Rabbit (local injection)	Postoperative pain	The nanofibers had good biocompatibility and eluted adequate levels of lidocaine and epinephrine during the initial stages of wound recovery	[62]
Lidocaine	Human epidermal growth factor (hEGF)	PLGA	Human foreskin fibroblasts/rat (lower abdominal implantation)	Surgical wound pain	The anti-adhesion nanofiber membrane with lidocaine and hEGF slow release had the effect of postoperative pain relief and wound healing	[63]
Baclofen	Lamotrigine	PLGA	Neuroblastoma cells/mouse leukemia cells of monocyte macrophage /rats and mice (intranasal injection)	Neuropathic pain	The system was a potential pro-inflammatory cytokine inhibitor, and the incidence of licking/biting in mice during inflammation-induced stage II pain significantly reduced	[64]
Dexamethasone	Diclofenac	PLGA	Mice (intraperitoneal injection)	Inflammatory pain	The water solubility of the system was enhanced, the drug loading reached 66%, the sustained release in vitro reached 52 h, and the maximum hydrolysis was achieved after 1.5 h. The anti-inflammatory activity showed a synergistic effect, and the inhibition rate of the TNF-α level was higher than that of the mother drug after 6 h	[65]
Dexamethasone	Celecoxib	Poloxamer 407/Gantrez S97-based gel system	Mouse leukemia cells of monocyte macrophage /male SD albino rats (intra-articular injection)	Knee OA	The system had good biocompatibility and a significant effect on inflammation. On day 8, the release of dexamethasone and celecoxib in the formulation was 98.6% and 92.2%, respectively, indicating a sustained therapeutic effect. It not only inhibited the release of TNF-α and IL-6 but also blocked cartilage surface flaking and matrix loss	[66]
Ropivacaine	Dexamethasone	Chitosan thermogel	SD rat (incision into the biceps femoris muscle of the right hind limb)	Postoperative pain	The system could limit sensory and motor function for up to 48 h, which had a significant effect on postoperative pain reduction	[67]
Ropivacaine	Dexamethasone	Hydrogel	L929 cells/SD rat (applied onto the epidermal surface of the skin)	Pain after musculoskeletal surgery	The anesthetic effect of co-loaded NPs with ropivacaine and dexamethasone was obviously better than that of hydrogel NPs without ropivacaine, and the anesthetic effect of ropivacaine could be increased by adding a small dose of dexamethasone	[68]
Dexibuprofen	Capsaicin	Emulgel	Franz diffusion cell/SD rat (applied topically to the right hind paw)	Foot edema	The emulsion effectively inhibited the foot edema induced by carrageenan and had higher analgesic activity	[69]
MTX	PEITC	Hydrogel	Rat (intra-articular injection)	RA	The preparation achieved good anti-inflammatory activity and reversed cartilage destruction through the synergistic effect between the two NP forms of MTX and PEITC, thus effectively improving the defects of its free form	[70]
Δ9-THC	Cibinetide	MSNs	Primary murine microglia cells/mice	Neuropathic pain	In vitro and in vivo experiments showed that the drug delivery system had the safety and effectiveness of reducing neuroinflammation, providing evidence for the feasibility of application in treating neuropathic pain	[71]
Tizanidine	Meloxicam	Bilayer mucoadhesive films	Male albino rabbits (intramuscular injection)	Postoperative pain	The absorption of drugs by oral mucosa was rapid, and the half-life was enhanced, showing the dual effect of the timely release of one drug and the continuous release of another drug	[72]
Ketorolac	Lidocaine	Polymeric wafer	Patients	Postoperative pain	The developed ketorolac acid/lidocaine polymer tablets proved effective in reducing pain and discomfort after gingival resection and in promoting wound healing	[73]

## 5. Nanoformulations Combined with Photothermal Therapy for Pain Treatment

Besides the aforementioned dual-drug combination therapy for pain, the combination of nanodrug delivery involving analgesic and photothermal therapy (PTT) has gradually become a promising treatment model. Tissue overheating and damage may occur during PTT, inevitably causing pain to patients. Therefore, painless PTT has become one of the important treatment goals in clinical practice [74]. We introduced some nanodrug delivery analgesics to relieve pain caused by PTT.

**Figure 6:**
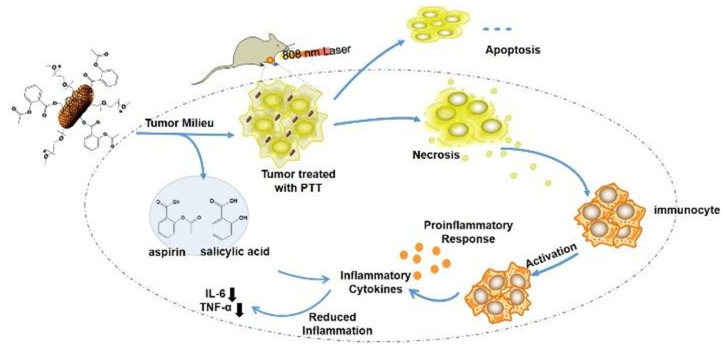
A schematic illustration of the anti-inflammatory mechanism underlying the inhibition of PTT-associated inflammation by gold NP complexes [77]. Copyright 2018 Wiley.

Jiang et al. proposed a treatment strategy to address the issue of chronic wounds, impaired angiogenesis, persistent pain, and increased inflammation while treating diabetic ulcers. They constructed a hydrogel system containing the photothermal agent black phosphorus with near-infrared light response characteristics. The hydrogel system generated heat and mediated the release of lidocaine hydrochloride under near-infrared light irradiation, thus alleviating the pain caused by PTT and reducing inflammation [75]. Black phosphorus could be degraded into nontoxic phosphate after exerting its function, which had unparalleled advantages in biomedical fields. Zhang et al. used the hydrogel doped with indocyanine green as the PTT photothermal agent and then loaded ropivacaine. The pain induced by PTT was accompanied by the activation of transient receptor potential vanilloid type-1 due to the increase in temperature. Compared with opioid analgesia, introducing local anesthetic ropivacaine into the hydrogel loaded with indocyanine could alleviate the pain caused by PTT and play a long-lasting analgesic role, providing a new understanding of analgesia and increasing photothermal therapy [76]. Dong et al. developed a treatment strategy that could provide enhanced PTT while inhibiting PTT-induced inflammatory response. The prodrug of aspirin was loaded onto graphite nanocapsules encapsulated in photothermal gold nanorods through π–π interactions. This composite nanomaterial was used to release aspirin in tumor environments while inhibiting PTT-induced inflammation. This strategy demonstrated good results both in vitro and in vivo and had profound implications for painless PTT (Figure 6) [77].

## 6. Conclusions and Future Perspectives

Pain management is one of the most important medical issues. Although using analgesics and anti-inflammatory drugs has reduced suffering over the past few decades, numerous limitations still exist. The nanomedicine delivery technology has gradually matured with the development of nanomedicine. Whether it is a single-drug carrier or a combination of multiple drugs, it has obvious advantages in pain treatment in terms of bioavailability, biosafety, pharmacokinetic characteristics, and other aspects. Although nanodrug delivery systems have created new perspectives for treating pain, they still have many undeniable limitations. (1) Nanoformulations reduced the side effects and improved the therapeutic effects of analgesics. However, the biosafety of nanomaterials used in the nanoformulations has not yet been fully evaluated, especially for inorganic nanomaterials. Long-term toxicity experiments and toxicity experiments on primates should be performed. (2) The improved bioavailability, pharmacokinetics, and therapeutic effects need further confirmation with beagles or primates. (3) Lack of data support for the stability and large-scale preparation of nanoformulations, which may also limit their clinical application. (4) Most nanomaterial-based drug delivery systems are injection preparations. For patients with chronic pain, oral preparations are more favorable.

Huge challenges exist in translating these advances into clinical practice. Initial evaluation of nanodrug delivery systems in extensive in vivo clinical studies is needed to obtain detailed information about the pharmacological effects of the dosing material in terms of efficacy to determine dosage and toxicity levels and predict possible adverse reactions. We hope that more efficient, safe, and functional intelligent nanodrug delivery systems can be developed and applied to clinical treatment, which can become a powerful tool for human beings to overcome pain.

## Data Availability

No new data were created.

## Abbreviations

NPs	Nanoparticles
IASP	International Association for the Study of Pain
RA	Rheumatoid arthritis
CBD	Cannabidiol
BCP	Beta-caryophyllene
NSAIDs	Nonsteroidal anti-inflammatory drugs
COX	Cyclooxygenase
MTX	Methotrexate
OA	Osteoarthritis
DAMGO	[D-Ala2, NMe-Phe4, Gly5-ol]-enkephalin
N/OFQ	Nociception/orphanin FQ
NOP	N/OFQ receptor
SLNs	Solid lipid nanoparticles
NLCs	Nanostructured lipid carriers
PAMAM	Poly(amidoamine)
PLGA	Poly(lactic-co-glycolic acid)
PEG	Polyethylene glycol
MSNs	Mesoporous silica nanoparticles
DOPr	δ-opioid receptor
SD	Sprague Dawley
CEL	Celonol
INdo	Indomethacin
IL-1ꞵ	Interleukin-1ꞵ
TNF-α	Tumor necrosis factor-α
TAT	Transactivator of transcription
PEITC	Phenethyl isothiocyanate
Δ9-THC	Δ9-tetrahydrocannabinol
hEGF	Human epidermal growth factor
PTT	Photothermal therapy
